# Enhancing the Interaction of Carbon Nanotubes by Metal–Organic Decomposition with Improved Mechanical Strength and Ultra-Broadband EMI Shielding Performance

**DOI:** 10.1007/s40820-024-01344-1

**Published:** 2024-02-27

**Authors:** Yu-Ying Shi, Si-Yuan Liao, Qiao-Feng Wang, Xin-Yun Xu, Xiao-Yun Wang, Xin-Yin Gu, You-Gen Hu, Peng-Li Zhu, Rong Sun, Yan-Jun Wan

**Affiliations:** 1 Shenzhen Institute of Advanced Electronic Materials, Shenzhen Institutes of Advanced Technology, Chinese Academy of Sciences, Shenzhen, 518055 People’s Republic of China; 2 Southern University of Science and Technology, Shenzhen, 518055 People’s Republic of China; 3 National Key Laboratory of Materials for Integrated Circuits, Shanghai Institute of Microsystem and Information Technology, Chinese Academy of Sciences, Shanghai, 200050 People’s Republic of China

**Keywords:** EMI shielding, Mechanical strength, Carbon nanotubes, Metal–organic decomposition, Flexibility

## Abstract

The remarkable properties of carbon nanotubes (CNTs) have led to promising applications in the field of electromagnetic interference (EMI) shielding. However, for macroscopic CNT assemblies, such as CNT film, achieving high electrical and mechanical properties remains challenging, which heavily depends on the tube–tube interactions of CNTs. Herein, we develop a novel strategy based on metal–organic decomposition (MOD) to fabricate a flexible silver–carbon nanotube (Ag–CNT) film. The Ag particles are introduced in situ into the CNT film through annealing of MOD, leading to enhanced tube–tube interactions. As a result, the electrical conductivity of Ag–CNT film is up to 6.82 × 10^5^ S m^−1^, and the EMI shielding effectiveness of Ag–CNT film with a thickness of ~ 7.8 μm exceeds 66 dB in the ultra-broad frequency range (3–40 GHz). The tensile strength and Young’s modulus of Ag–CNT film increase from 30.09 ± 3.14 to 76.06 ± 6.20 MPa (~ 253%) and from 1.12 ± 0.33 to 8.90 ± 0.97 GPa (~ 795%), respectively. Moreover, the Ag–CNT film exhibits excellent near-field shielding performance, which can effectively block wireless transmission. This innovative approach provides an effective route to further apply macroscopic CNT assemblies to future portable and wearable electronic devices.

**Supplementary Information:**

The online version contains supplementary material available at 10.1007/s40820-024-01344-1.

## Highlights

- A strategy based on metal-organic decomposition is proposed to enhance the tube-tube interactions of carbon nanotubes (CNTs).
- The robust tube-tube interactions of CNTs enhance both EMI shielding performance and mechanical properties of CNT film.
- This innovative approach provides an effective way to obtain high-performance CNT film.

**Supplementary Information:**

The online version contains supplementary material available at 10.1007/s40820-024-01344-1.

## Graphical abstract

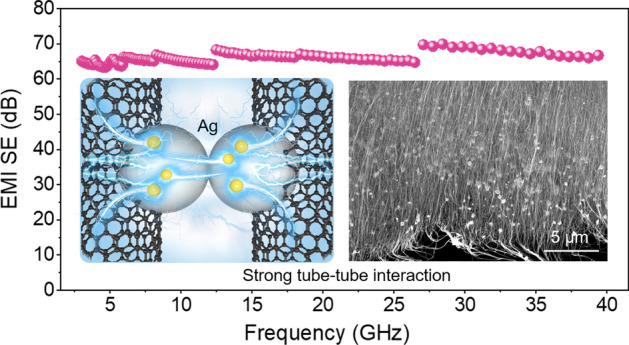

## Introduction

The proliferation of portable devices and wireless communication has resulted in an increasingly severe problem of electromagnetic (EM) radiation, which adversely impacts both electronic devices and living beings [1–4]. The development of efficient materials for electromagnetic interference (EMI) shielding with ultra-broadband, high mechanical stability, flexibility, and ease of manufacture has become crucial [5–8]. Carbon-based materials have attracted widespread attention because of their unique advantages, such as lightweight, flexibility, and good chemical stability [9–11]. Notably, carbon nanotubes (CNTs) stand out for their outstanding electrical conductivity, high mechanical strength, and ability for mass production, demonstrating enormous potential in the development of EMI shielding materials. The electrical conductivity of a single CNT is as high as 1 × 10^8^ S m^−1^, and the mechanical strength exceeds 100 GPa, owing to its strong C=C bond and large π-conjugated system [12]. Unfortunately, these excellent characteristics are still elusive in their macroscopic assemblies, such as CNT films.

The most significant factor causing this disappointing difference is the weak tube–tube interactions due to discontinuity, misalignment, and loose stacking of the CNT film [13, 14]. The loose structure exhibits high tunneling barriers between conducting regions and large contact resistance between CNT tubes, which significantly affects the transport of charge carriers among CNT tubes [15–17]. Similarly, the components in the CNT film cannot bear the load synchronously and uniformly, limiting improvement in their overall tensile strength [18]. Aside from controlling the distance between CNT tubes, the introduction of stable connections among tubes has also been proven to be an effective way to obtain high-performance CNT film. It has been reported that the difference in Fermi energy levels between metal and CNT promotes electron transport in the CNT tubes, reducing the energy required for electrons to pass through the potential barrier [19]. The introduction of metal particles helps to improve the electrical properties of CNT film, showing obvious advantages in reinforcing the intertube connections of CNT film. Therefore, we rationally speculated that the introduction of silver (Ag) particles would result in stronger tube–tube interactions and higher conductivity, potentially leading to more suitable EM parameters and thus improving the EMI shielding performance of the CNT film. Regarding mechanical properties, it has been reported that metal particles can fix discrete CNTs firmly with controlled intertube sliding, leading to a more uniform strain distribution [18, 20, 21]. Thus, more efficient intertube load transfer and higher tensile strength of CNT film can be achieved. Inspired by these considerations, we envisage that it is desirable to optimize the tube–tube interactions by introducing metal particles into CNT film to improve both the EMI shielding performance and mechanical strength. Unfortunately, it is difficult for metal particles to enter the CNT film due to the chemically inert graphite surface and hydrophobicity characteristic of CNTs [22]. To the best of our knowledge, few studies have reported the introduction of metal particles within CNT film. Therefore, it is important to explore efficient tactics to address these limitations.

Metal–organic decomposition (MOD) is composed of ionic metals and involves metal precursors that are simply dissolved in suitable solvents or use organic complexing agents as solvents [23]. It does not rely on the presence of metal particles in the solution. In this work, we developed a novel strategy based on MOD to fabricate a high-performance Ag–CNT film. The Ag particles are introduced in situ into the CNT film through annealing of MOD, leading to enhanced tube–tube interactions. The obtained Ag–CNT film shows outstanding flexibility, improved mechanical strength (76.06 ± 6.20 MPa) and electrical conductivity (6.82 × 10^5^ S m^−1^). The EMI shielding effectiveness (SE) of Ag–CNT film with a thickness of only ~ 7.8 μm is as high as 66 dB in the ultra-broadband frequency range (3–40 GHz). Moreover, Ag–CNT film exhibits excellent near-field shielding performance, which can effectively block wireless power transmission. Our approach provides a new method for producing high-quality flexible shielding materials, which is critical to the development of a variety of applications.

## Experimental Section

### Materials

Silver acetate (CH_3_COOAg, AR 99.5%), ethylenediamine (C_2_H_8_N_2_, GC > 99.0%), and formic acid (CH_2_O_2_, AR 88.0%) were purchased from Aladdin. Ammonium hydroxide (NH_3_·H_2_O, ~ 25% NH_3_ basis) and ethanol (C_2_H_6_O, AR 99.0%) were provided by Sinopharm Chemical Reagent Co., Ltd., China. CNT films were purchased from Suzhou Jiedi Nanotechnology Co., Ltd., China.

### Synthesis Process of MOD

Silver acetate powders (2.5, 5, and 10 g) were first mixed with ethylenediamine (25 mL) in a glass beaker by electromagnetic stirring until the mixture cooled naturally and then slowly add ammonium hydroxide. Subsequently, formic acid (2 mL) diluted with ethanol was dropped into the mixed solution. Various concentrations (0.25, 0.5, 1 mol L^−1^) of MOD were obtained by centrifugation at 8000 rpm to filter the solution based on organic Ag precursor.

### Preparation Process of Ag–CNT Films

The CNT film underwent oxygen (O_2_) plasma treatment to improve the interaction between Ag and CNTs. Ag–CNT film was prepared by ‘absorption–annealing’ process. The MOD was dropped into the pre-treated CNT film and subsequently absorbed. After annealing under a nitrogen atmosphere within a tube furnace at 200 °C for 10 min, an in situ reduction of Ag particles occurred within the CNT film. Various concentrations of MOD were utilized in the ‘absorption–annealing’ process to yield Ag–CNT films with different Ag contents. CNT films with Ag content of 42, 51, and 66 wt% were denoted as Ag–CNT film-1, Ag–CNT film-2, and Ag–CNT film-3, respectively.

### Characterization and Test Methods

The morphology and microstructure of Ag–CNT film were examined using a scanning electron microscope operating at 10 kV accelerating voltage (SEM, Nova Nano SEM450, FEI). Elemental compositions of Ag–CNT film were analyzed through a SEM equipped with energy-dispersive X-ray spectroscopy (EDS) and X-ray photoelectron spectroscopy (XPS, ESCALAB 250 Xi, Thermo Fisher). Structural analyses were performed using X-ray diffraction (XRD) measurements with a D8 Advance X instrument. Raman spectra were collected by inVia (Renishaw, Britain). Thermogravimetric analysis (TGA) was carried out employing a TA Instruments Q600 at a temperature rate of 10 °C min^−1^ under an air atmosphere. Mechanical properties were assessed using DMA850 (TA Instruments), with a minimum of five samples tested. Electrical conductivity was measured using a four-pin probe (PSP, MCP-TP06P). EMI SE in the frequency range covering the 2.6–3.95 GHz (S-band), 3.94–5.99 GHz (C-band), 5.38–8.17 GHz (C-band), 8.2–12.4 GHz (X-band), 11.9–18 GHz (Ku-band), 18–26.5 GHz (K-band), and 26.3–40 GHz (Ka-band) was evaluated employing a vector network analyzer (VNA, Keysight, E5071C) using the waveguide method. The sample with a suitable size was positioned on calibrated waveguide holders for testing. Total EMI SE (SE_T_) its reflection (SE_R_), and absorption (SE_A_) are calculated by the following equations [24, 25]:

1 $$R = \frac{{P_{{\text{R}}} }}{{P_{{\text{I}}} }} = \left| {S_{11} } \right|^{2}$$

2 $$T = \frac{{P_{{\text{T}}} }}{{P_{{\text{I}}} }} = |S_{21} |^{2}$$

3 $$A = 1 - R - T = 1 - \left| {S_{11} } \right|^{2} - |S_{21} |^{2}$$

4 $${\text{SE}}_{{\text{R}}} \left( {{\text{dB}}} \right) = - { }10\log \left( {1 - \left| {S_{11} } \right|^{2} } \right)$$

5 $${\text{SE}}_{{\text{A}}} \left( {{\text{dB}}} \right) = - { }10\log \left( {\frac{{|S_{21} |^{2} }}{{1 - \left| {S_{11} } \right|^{2} }}} \right)$$

6 $${\text{SE}}_{{\text{T}}} \left( {{\text{dB}}} \right) = {\text{SE}}_{{\text{R}}} + {\text{SE}}_{{\text{A}}}$$

where *R* is the reflection coefficient, *T* is the transmission coefficient, and *A* is the absorption coefficient. *P*_I_ is incoming power, *P*_R_ is reflected power, and *P*_T_ is transmitted power. The EMI SE test at low frequency (30 MHz–1.5 GHz) was conducted following ASTM D4935-99 standards, employing a standard enlarged coaxial transmission line sample holder (KEYCOM, Japan). Near-field shielding performance was measured using the Smart Scan-350/550 EMI system (API, UK).

## Results and Discussion

### Fabrication Process of Ag–CNT Films

**Fig. 1:**
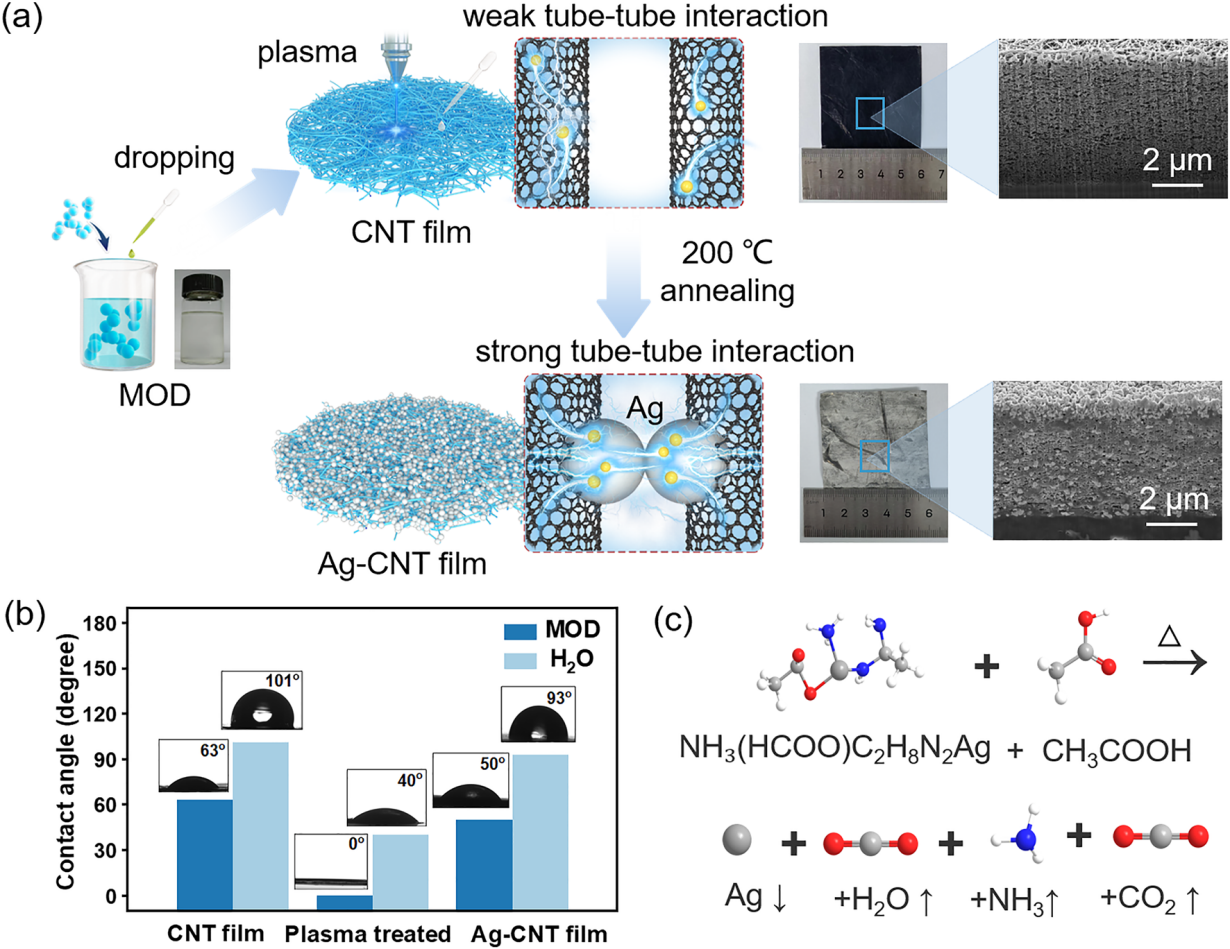
**a** Schematic of the process of introducing Ag particles into the interior of CNT film. The introduction of Ag particles enhances the tube–tube interactions. **b** Contact angles of the film at different stages. **c** Chemical reaction equation for the thermal decomposition of MOD

The process of introducing Ag particles into the CNT film to enhance tube–tube interactions is illustrated in Fig. 1a. The CNT film appears black and demonstrates hydrophobicity with a contact angle of 101° (Fig. 1b). This property poses a challenge for the integration of foreign materials with CNTs [26, 27]. Notably, the MOD exhibits a smaller contact angle of 63° when interacting with CNT film, yet complete penetration is not achieved. To further improve the wettability of CNTs and promote a robust bond with metal particles, plasma treatment was applied to modify the surface of CNT film.

This process induces the creation of oxidized vacancies and graft oxygen-containing functional groups (e.g., C=O, C–O–C) [28], as confirmed by Raman spectrum results (Fig. S1). Typically, these vacancies can trap metal atoms, serving as nucleation centers and facilitating the formation of Ag particles [26, 29]. Without oxygen plasma treatment, there is only a minimal amount of silver-amine complex formed by the coordination of Ag^+^ with nitrogen on the surface of CNT film [30], and there is almost no penetration into the interior of CNT film. Upon careful dropping the MOD onto the CNT film with plasma treatment, the contact angle with MOD significantly decreases to 0° due to the enhanced hydrophilicity. Consequently, the silver-amine complex can effectively penetrate into the CNT film and be absorbed by the completely wetted CNT. Following in situ annealing of the MOD-CNT film at 200 °C (Fig. S2), the silver-amine complex is reduced to form Ag particles. The obtained Ag–CNT film shows a silver color and exhibits an overall metallic luster (Fig. 1a). As depicted in Fig. 1c, during the thermal decomposition process, volatile gases are generated without leaving residues that might impede the electrical conductivity of the CNT film. A higher degree of Ag particle embedding occurs on the surface and inside CNT film with plasma treatment, compared to untreated film (Fig. S3). The van der Waals force between Ag particles and CNTs [26] effectively forms a bridge-like structure between adjacent tubes. This connection optimizes the tube–tube interactions of CNT film, significantly enhancing its mechanical strength, electrical conductivity, and EMI shielding performance.

### Characterization Analysis of Ag–CNT Films

**Fig. 2:**
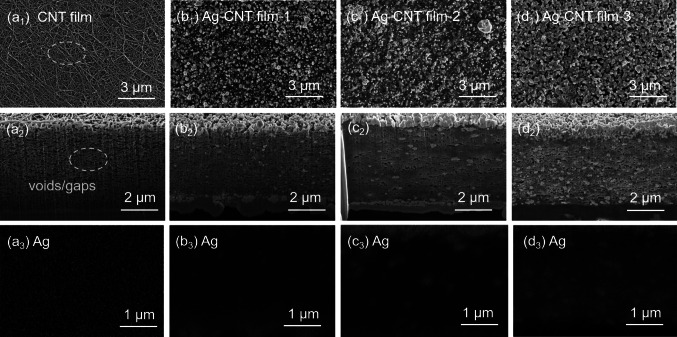
Morphologies of CNT films before and after the introduction of Ag particles.** a**_**1**_–**d**_**1**_ SEM images of the surface morphology. **a**_**2**_–**d**_**2**_ SEM images of the cross-sectional morphology, showing the introduction of Ag particles within the voids and gaps of CNT film. **a**_**3**_–**d**_**3**_ The corresponding EDS maps, displaying the presence of the Ag element

The microstructure of CNT film and Ag–CNT films with varying Ag contents was characterized via SEM, as demonstrated in Fig. 2. The CNT film exhibits loosely interconnected network structures, as numerous voids and gaps between CNT tubes can be observed (Fig. 2a_1_, a_2_), which adversely impact the tube–tube interactions [13]. After the coating of MOD and annealing process, MOD was thermally reduced to Ag particles and in situ formed both on the surface and inside of CNT film. Notably, the amount of Ag particles introduced into the CNT film can be easily controlled by only adjusting the MOD concentration. As shown in Fig. 2b1, b2, when the MOD concentration is low (0.25 mol L^−1^), only a small amount of Ag particles is achieved and sparsely dispersed inside the CNT film. Few Ag particles are insufficient to establish an effective tube–tube connection, thus posing limitations on the overall performance improvement of the CNT film. With a gradual increase in the concentration of the MOD, the amount of Ag particles inside the CNT film was progressively increased, while the Ag particles on the surface of the CNT film were gradually saturated. When the MOD concentration is 0.5 mol L^−1^, part of the voids and gaps are filled with Ag particles (Fig. 2c_1_, c_2_). The voids and gaps in CNT film are almost filled with Ag particles when the MOD concentration is up to 1 mol L^−1^ (Fig. 2d1, d2). These Ag particles are effectively interconnected with adjacent tubes, forming a densely interconnected tube network. And the structures of CNTs are well maintained after the annealing process. The results of Ag element mapping shown in Fig. 2a_3_–d_3_ further confirm the increase of Ag content inside CNT film with the increased MOD concentration. By employing the MOD with thermal annealing method, we successfully introduced Ag particles into the voids/gaps of CNT film, which facilitates the achievement of robust tube–tube interactions, thereby enhancing both the mechanical properties and electrical conductivity of CNT film.

**Fig. 3:**
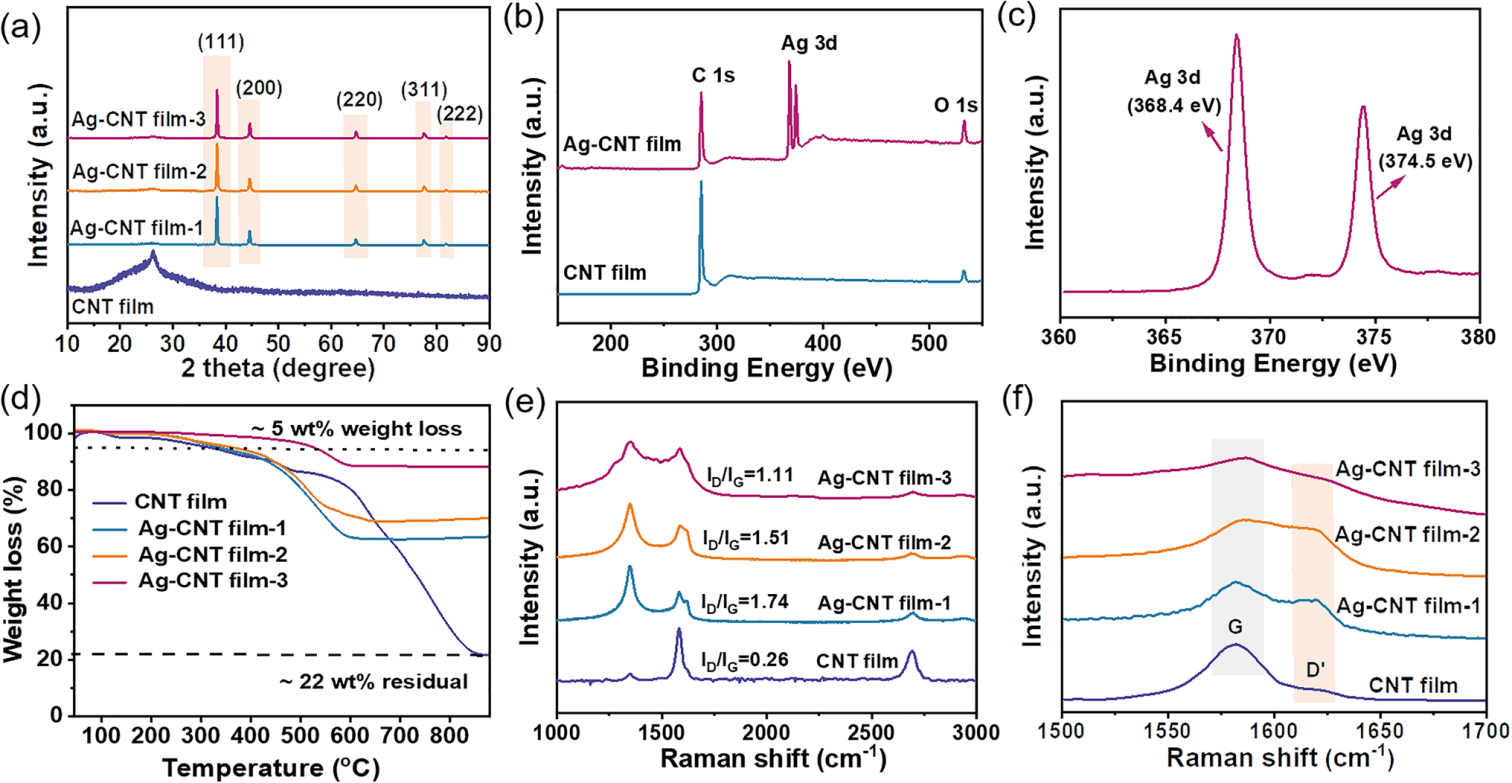
**a** XRD patterns, showing the appearance of Ag characteristic peaks in the Ag–CNT films. **b** XPS spectra of films. **c** High-resolution XPS spectrum of Ag 3*d* in the Ag–CNT film. **d** TGA results of the films with different Ag contents (~ 42, 51, and 66 wt%). **e** Raman spectra of the CNT film and Ag–CNT films. **f** Strong *G* peak with a small shoulder *D*′ peak that weakens with increasing Ag content

To elucidate the crystal structure and elemental composition of both CNT film and Ag–CNT films, XRD and XPS analyses were conducted. As depicted in Fig. 3a, the CNT film exhibits a characteristic peak of carbon *s*at 2*θ* = 26.5°. Following the process of introducing Ag particles, the discernible carbon characteristic peak almost vanishes. This phenomenon is possibly attributed to the Ag particles obscuring the signals from CNT. For Ag–CNT film, there are additional sharp peaks located at 2*θ* = 37.98°, 44.17°, 64.32°, 77.27°, and 81.44°, respectively, which can be categorized as the face-centered cubic phase of Ag [31, 32].

Figure 3b displays the C 1*s* and O 1*s* peaks derived from the CNT, and the new characteristic Ag 3*d* peaks appeared in the Ag–CNT films. The high-resolution XPS spectrum of Ag 3*d* in Ag–CNT film is presented in Fig. 3c. The peaks are located at 368.4 and 374.5 eV, corresponding to Ag 3*d*_5/2_ and Ag 3*d*_3/2_, respectively, which further confirms the successful introduction of Ag particles onto CNTs. TGA was conducted to quantify the Ag content, as shown in Fig. 3d. The result illustrates that CNT film residue is approximately 22 wt% as the temperature exceeded 800 °C. This residue is attributed to an iron-based catalyst, as confirmed by the EDS mapping (Fig. S4). By calculating the weight difference of residue between CNT film and Ag–CNT films from TGA results, the Ag contents in Ag–CNT films are determined to be 42, 51, and 66 wt% for Ag–CNT film-1, Ag–CNT film-2, and Ag–CNT film-3, respectively. It is interesting to note that the thermal stability of the Ag–CNT film is improved with increased Ag content, which has a higher thermal degradation temperature (ca. 527 °C in air). Furthermore, Raman spectroscopy was employed to unveil the microstructural characterizations and quality of CNT film before and after the introduction of Ag particles [33, 34]. The peak intensity ratio *I*_D_/*I*_G_ between the D (~ 1350 cm^−1^) and G (~ 1580 cm^−1^) bands, as shown in Figs. 3e and S5a, indicates a gradual reduction in *I*_D_/*I*_G_ after the process of introducing Ag particles. This reduction may be attributed to the annealing process gradually removing the oxygen-containing groups grafted on the CNTs [35]. As depicted in Figs. 3f and S5b, the intensity of D’ peaks in the Ag–CNT films, corresponding to the degree of response to *E*_2g_ in-plane vibration, is weakened with the increased Ag content when compared with that of the CNT film with plasma treatment. This phenomenon is in part because of the enhanced tube–tube interactions facilitated by Ag particles, which reduces the vibration of suspended bonds of CNT, consistent with our previous studies [13].

### Mechanical Properties of Ag–CNT Films

**Fig. 4:**
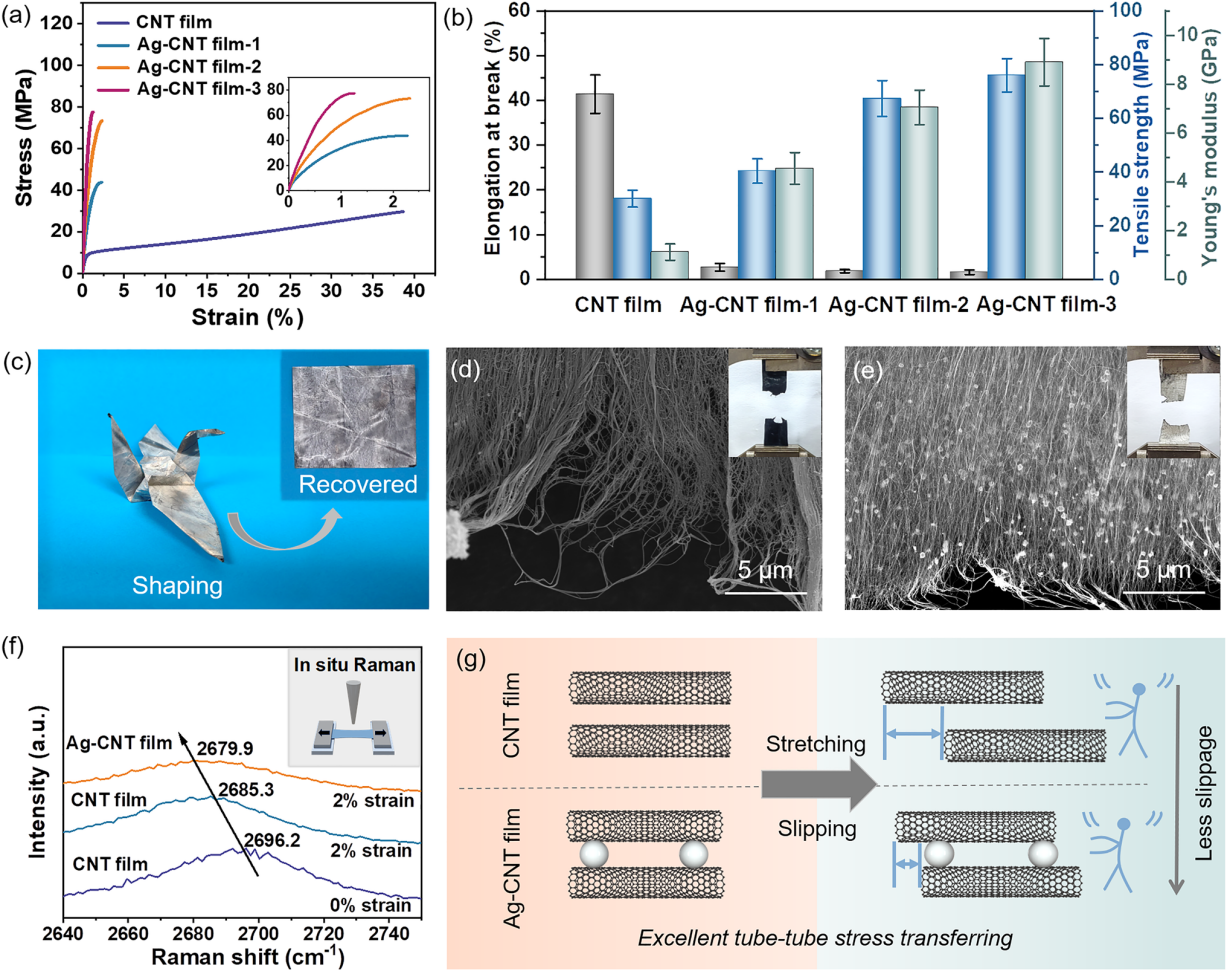
Mechanical properties of CNT films before and after the introduction of Ag particles: **a** stress–strain curves (the illustration is the stress–strain curves of Ag–CNT films, exhibiting high tensile strength). **b** Summarization of the tensile strength, elongation at break, and Young’s modulus. **c** Optical images of Ag–CNT film showing its flexibility. Optical images of the fracture edge of **d** CNT film and **e** Ag–CNT film. **f** Corresponding Raman shift changes of G’ band at 2% strain. **g** Schematic of excellent tube–tube stress transfer-induced improved mechanical properties of the Ag–CNT film

The introduction of Ag particles into CNT films not only preserves their flexibility but also enhances their mechanical properties significantly. The stress–strain curves of CNT film and Ag–CNT films, along with the summarized tensile strength, elongation at break, and Young’s modulus are shown in Figs. 4a, b and S6. The CNT film owns a tensile strength of 30.09 ± 3.14 MPa and an elongation at break of (41.39 ± 4.30)%. With the introduction of Ag particles, the Ag–CNT films exhibit a substantial improvement in tensile strength. The tensile strength of Ag–CNT film-1 and Ag–CNT film-2 is increased to 40.38 ± 4.56 and 71.52 ± 7.42 MPa, while the elongation at break is decreased to (2.73 ± 0.87)% and (2.06 ± 0.73)%, respectively. Particularly, the tensile strength of Ag–CNT film-3 is further increased to 76.06 ± 6.20 MPa, which is 253% higher than that of CNT film, and the elongation at break is only (1.63 ± 0.50)%. Meanwhile, the Young’s modulus of films was calculated from stress–strain curves (Table S1). When the Ag content is increased, the Young’s modulus of Ag–CNT film-3 shows an extraordinary increase of 795% in comparison with that of CNT film (from 1.12 ± 0.33 to 8.90 ± 0.97 GPa). Moreover, Ag–CNT film can be easily folded into complex shapes and has no structural disintegration after unfolding (Fig. 4c), further demonstrating excellent flexibility. The SEM image of the fracture surface (Fig. 4d) reveals a loose structure in the CNT film, and some pulled-out CNTs can be observed. However, the Ag–CNT film shows a compact fracture edge due to the embedded Ag particles between CNTs (Fig. 4e), verifying the improved tube–tube interactions in the Ag–CNT film.

As it is known, the interaction between CNTs has a particularly critical impact on the mechanical properties of macroscopic CNT assemblies. During the stretching process, the components of CNT film start to slide due to the unsynchronized and uniform loading. With the increase of strain, CNT is further stretched in the tensile direction until a fracture occurs [36, 37]. Metal particles can fix discrete CNTs, limiting slippage between CNTs, which plays a unique role in maintaining tube–tube connections and facilitating load transfer between CNTs [18]. It is expected that embedding Ag particles in CNT films will achieve more efficient tube–tube load transfer, improve CNT tube–tube interactions, and ultimately enhance the tensile strength of CNT film.

To further validate above result, in situ Raman spectra of films during tensile deformation were recorded using a home-made device (Fig. S7). The G’ peak, located at 2696.2 cm^−1^, is related to the *sp*^*2*^*–sp*^*2*^ bond in CNTs and it is sensitive to stress, which is typically used to detect stress in CNTs [38]. Notably, the G’ peak of Ag–CNT films shifts to a lower wavenumber (from 2685.3 to 2679.9 cm^−1^) under tensile strain of 2% (Fig. 4f). This should be attributed to the decrease in vibration frequency resulting from the increased *sp*^*2*^–*sp*^*2*^ bond length in CNTs with increased tensile strain [39–41]. This result demonstrates that due to the introduction of Ag particles, Ag–CNT films exhibit a more uniform strain distribution and more efficient stress transfer between CNTs under the identical strain. The embedded Ag particles in CNT film can effectively prevent the slippage between CNTs (Fig. 4g), as confirmed by the reduced elongation at break of Ag–CNT film. These collective effects synergistically result in the improved mechanical properties of Ag–CNT films.

### EMI Shielding Performance of Ag–CNT Films

**Fig. 5:**
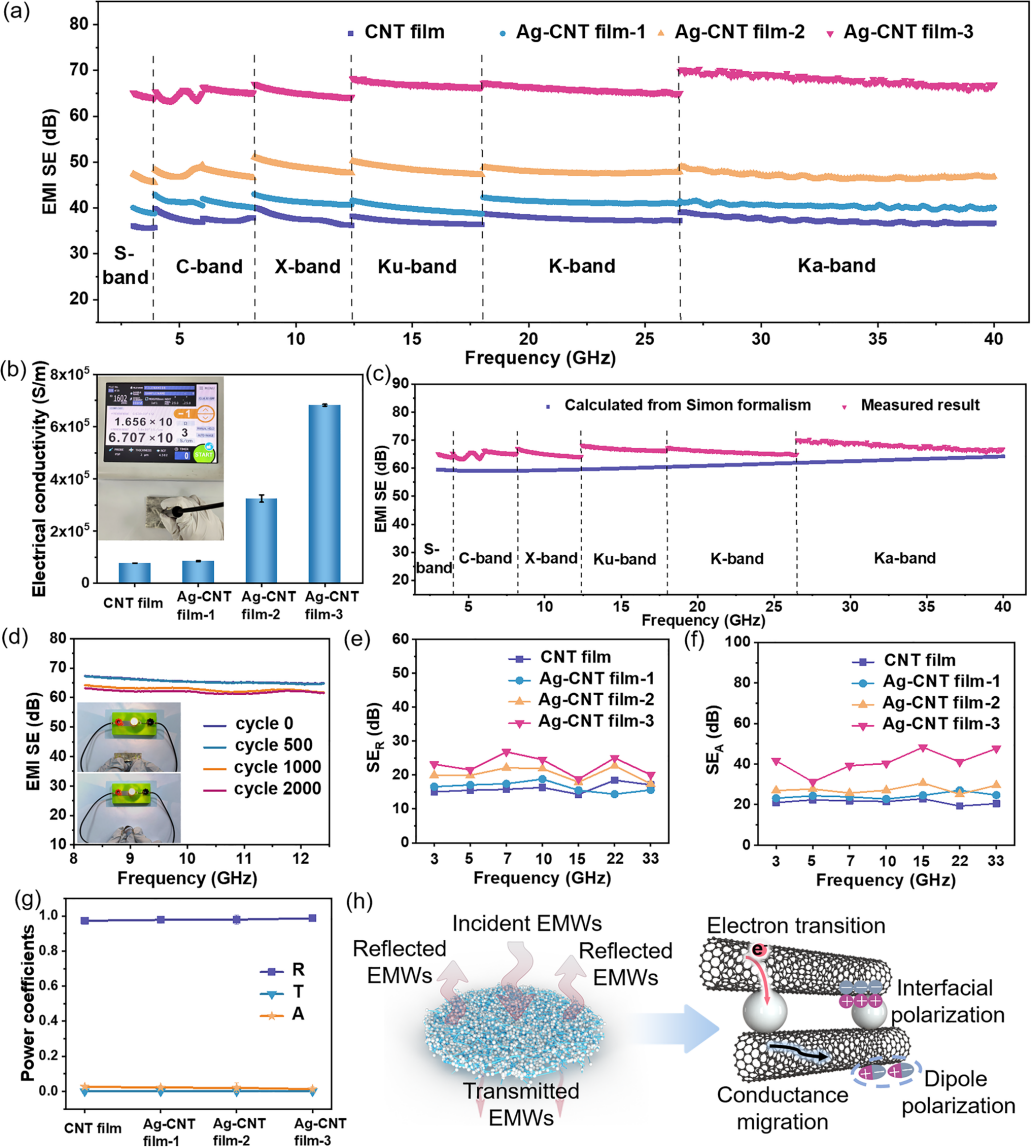
EMI shielding performance of CNT film and Ag–CNT films in an ultra-broadband frequency range. **a** EMI SE of the films in the frequency range of 3–40 GHz. **b** Electrical conductivity of the films. **c** Comparison of experimental and theoretical (calculated from Simon’s formalism) EMI SE. **d** X-band EMI SE of Ag–CNT film-3 after 2000-cycle bending (bending angle *θ* = 180°). EMI shielding performance, including average **e** SE_R_ and** f** SE_A_ in the frequency of 3–40 GHz for films with different Ag content. **g** Comparison of power coefficients of *R*, *A*, and *T* values for films with different Ag contents. **h** EMI shielding mechanism of the Ag–CNT film

The EMI SE of the Ag–CNT films with different Ag contents was measured in a wide frequency range of 3–40 GHz, as demonstrated in Fig. 5a. The CNT film exhibits an average EMI SE of 37 dB with a thickness of 5 μm (Table S2). The introduction of Ag can effectively improve the shielding performance of CNT film, and EMI SE is easily controlled by adjusting the Ag content. The Ag–CNT film-1 and Ag–CNT film-2 possess EMI SE of 41 dB (at ~ 5.9 μm) and 48 dB (at ~ 6.5 μm), respectively. It is particularly noteworthy that the Ag–CNT film-3 achieves an extraordinary EMI SE of 66 dB with a thickness of ~ 7.8 μm, blocking over 99.9999% of incident radiation while only transmitting 0.0001%. The high EMI SE of Ag–CNT film-3 with thin thickness leads to ultrahigh specific SE (defined as SE divided by thickness) of 84,615.4 dB cm^−1^. Interestingly, the EMI SE of both CNT film and Ag–CNT films is slightly improved with the increase in frequency. This phenomenon may result from the significant skin effect of the highly conductive film.

The skin effect, defined as the phenomenon of EM radiation penetrating only the near-surface area of the electrical conductor at high frequencies [42], is influenced by the skin depth ($\delta$) given by $\delta =\frac{1}{\sqrt{\pi f\mu \sigma }}$[43], where $\sigma$ and $\mu$ are the electrical conductivity and magnetic permeability of the shield, respectively, and $f$ is the frequency. For non-magnetic materials with constant thickness, the skin depth is decreased with the increase in frequency [5], resulting in improved attenuation of total electromagnetic waves (EMWs) beneath the surface of the shielding film. According to Simon’s formula, the EMI SE can be expressed as: ${\text{SE}}=50+10{\text{log}}\left(\sigma /f\right)+1.7t\sqrt{\sigma f}$ [44, 45], where *f* (MHz),* σ* (S cm^−1^), and* t* (cm) are the electrical conductivity, frequency, and sample thickness, respectively. The EMI SE and electrical conductivity exhibit a proportional relationship, indicating that Ag–CNT film with higher electrical conductivity exhibits higher EMI SE (Fig. 5b). Compared with CNT film (~ 7.7 × 10^4^ S m^−1^), Ag–CNT film-1 has a comparable electrical conductivity (~ 8.5 × 10^4^ S m^−1^). With the further increase in Ag content, the electrical conductivity of Ag–CNT film-2 and Ag–CNT film-3 increases to 3.24 × 10^5^ and 6.82 × 10^5^ S m^−1^, respectively. The remarkable electrical properties primarily stem from the high conductivity of both Ag and single CNT, along with the robust tube–tube interactions among CNTs, which substantially facilitate electron transport and improve the electrical conductivity. The experimental results of Ag–CNT film-3 in the frequency range of 3–40 GHz are comparable to the theoretical calculation results based on Simon’s formula, as shown in Fig. 5c. In addition, the formula predicts high EMI SE values for Ag–CNT film-3 at lower frequencies (Fig. S8a). Measurement of EMI SE of Ag–CNT film-3 at low frequency (30 MHz–1.5 GHz) using a coaxial transmission line method confirmed this prediction, showing similar EMI SE values at high and low frequencies (Fig. S8). As a result, Ag–CNT film maintains excellent EMI shielding capability over an ultra-broadband frequency range. To demonstrate the flexibility of CNT film after embedded with Ag particles, the EMI SE of Ag–CNT film-3 with different bending cycles was measured (Fig. 5d), and the corresponding SEM images after bending are shown in Fig. S9. The Ag–CNT film-3 exhibits a negligible decay in EMI SE even after bending for 2000 cycles, which can be seen more intuitively by the negligible change in lamp brightness, as illustrated in Fig. 5d. The EMI SE of Ag–CNT film-3 retains 94% of the original EMI SE, and this excellent flexibility characteristic of Ag–CNT film may be contributed to the strong interaction between Ag and CNT.

EMI shielding results from a synergistic combination of reflection and absorption for EMWs. The SE_R_ and SE_A_ of Ag–CNT films are increased with the increase in Ag content. Significantly, the SE_A_ value improves substantially, from 21 to 41 dB, whereas the value of SE_R_ only shows a slight rise from 16 to 23 dB (Fig. 5e, f). This discrepancy should be attributed to the different growth rates resulting from distinct functional formulas based on conductivity in the calculation of SE_A_ and SE_R_ (see Eqs. S1–S3). To further analyze the shielding mechanism of Ag–CNT film, the A, R, and T are calculated by Eqs. (1)–(3), as shown in Fig. 5g. With the increase of Ag content, the R-value of Ag–CNT films is steadily increased to 0.99. The consistently higher R-value compared to A indicates a reflection-dominant shielding mechanism of Ag–CNT film, which is similar to the reported results [46–48]. As illustrated in Fig. 5h, when EMWs are incident on the surface of the Ag–CNT film, the majority of incident EMWs are reflected back, owing to the impedance mismatch between the highly conductive Ag–CNT film and the free space [43, 49]. The residual incident EMWs continuously penetrate into the Ag–CNT film for further attenuation by ohmic loss, and the EM energy is further converted into thermal energy [50]. Besides, under alternating EM fields, the interface polarization induced by the accumulation of free charges at the heterogeneous interfaces between Ag particles and CNTs, the dipole polarization caused by residual groups, as well as defects in Ag–CNT film acting as electric dipoles lead to higher losses to improve the overall EMI SE synergistically [51–53].

### Near-Field Shielding Performance and Application Demonstration of Ag–CNT Films

The EM radiation is divided into near-field radiation (*KR* ≪ 1) and far-field radiation (*KR* ≫ 1) based on the distance between the radiation source and the shielding material, where *K* and *R* are the wavenumber and distance from detector to radiation source, respectively [54]. The EMI of electronic devices is usually located in the near field of the emission source. Therefore, the near-field shielding performance test offers a more precise evaluation of the SE in practical applications [55, 56]. Near-field scanning technology enables direct measurement of the electrical and magnetic radiation intensity at a spatial point through the probe, acquiring the radiation near-field cloud [57].

**Fig. 6:**
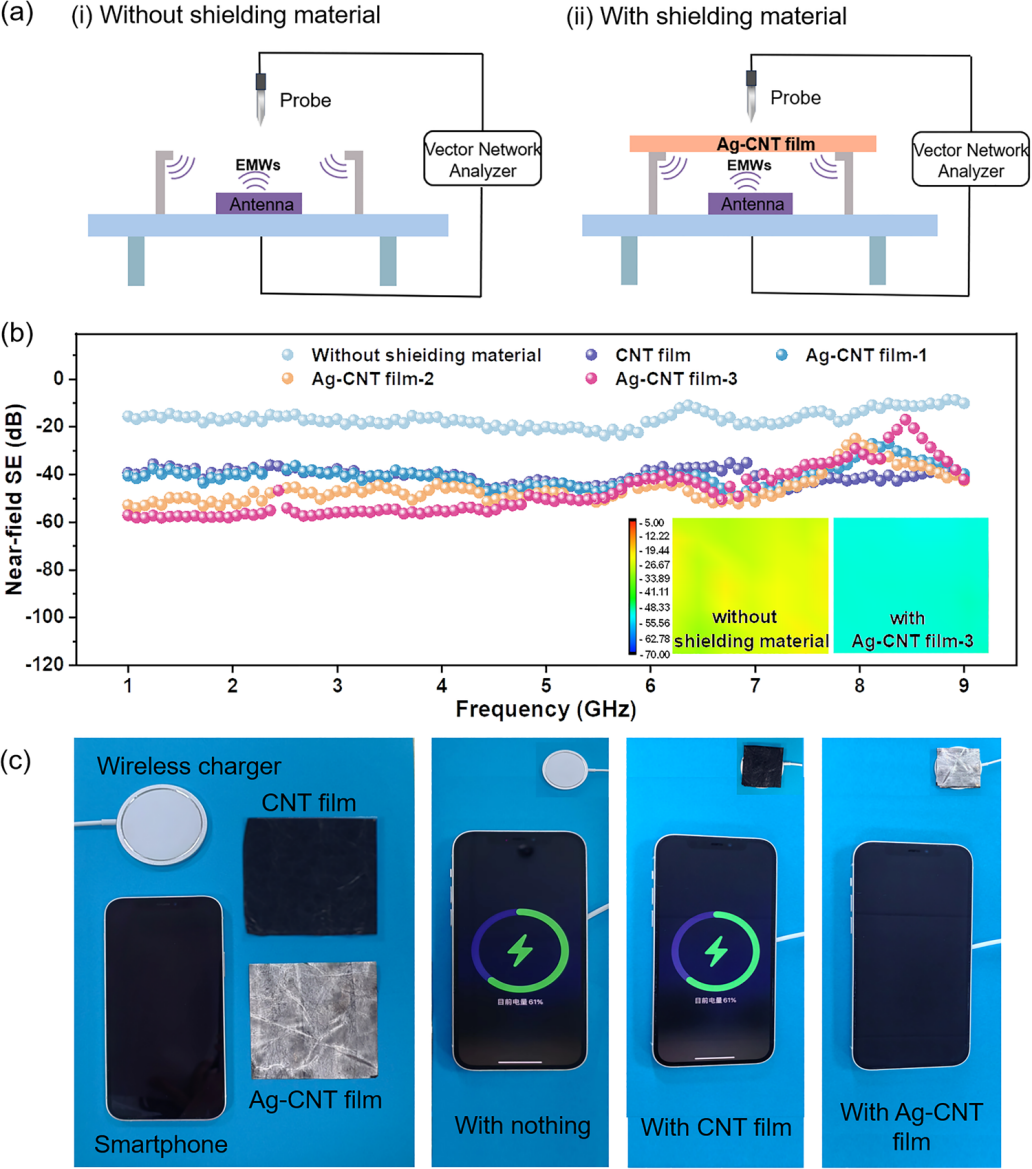
**a** The schematic for measurement of near-field SE **i** without and **ii** with shielding material. **b** The near-field SE of CNT film and Ag–CNT films in the frequency range of 1–9 GHz (inset is the 2D near-field SE mapping). **c** Demonstration of EMI shielding performance of Ag–CNT film for wireless charging

The schematic illustration of the test principle and the corresponding equipment is presented in Fig. 6a and Fig. S10, respectively. In our study, we measured the near-field SE of Ag–CNT film in the frequency range of 1–9 GHz, employing the microstrip antenna as the radiation source. As demonstrated in Fig. 6b, the near-field shielding performance of Ag–CNT film improves with the increase in Ag content. The near-field SE value of Ag–CNT film-1 is approximately − 41 dB, much higher than that of the baseline without shielding material (about − 16 dB). The near-field SE values of Ag–CNT film-2 and Ag–CNT film-3 further increase to − 46 and − 48 dB, respectively. The inset in Fig. 6b shows the visualized signal intensity of the leakage EMWs with or without shielding material. Radiation hot spots are concentrated when there is no shielding material, and this can be considered as antennas with strong vertical radiation. Therefore, the field distribution is more uniform when Ag–CNT film-3 is used as the shielding material. Moreover, the corresponding near-field SE mapping with Ag–CNT film-3 is gradually changed from yellow to light blue, indicating that the detected EM radiation is weakened. As a proof of concept to assess the EMI shielding performance of Ag–CNT film in practical conditions, the power transmission of mobile phones during wireless charging was observed (Fig. 6c). We placed nothing, CNT film, and the Ag–CNT film between the smartphone and wireless charger to evaluate the effect on charging. Obviously, the smartphone displays a normal charging status when covered with CNT film, similar to the uncovered state. However, it cannot be charged when it is covered with Ag–CNT film, demonstrating the effective blocking of wireless power transmission. The promising result suggests a bright outlook for the application of CNT films.

## Conclusions

In conclusion, we applied a new strategy based on MOD to introduce Ag particles into the CNT film, resulting in enhanced tube–tube interactions, which endowed CNT film with outstanding mechanical strength (76.06 ± 6.20 MPa), electrical conductivity (6.82 × 10^5^ S m^−1^), and ultra-broadband EMI shielding performance (66 dB in 3–40 GHz). Furthermore, the Ag–CNT film demonstrated remarkable near-field shielding performance, presenting a potential application in electronic packaging. Our finding provides a novel approach to obtaining high-performance CNT film, which paves the way for the industrial applications of CNT films.

## Supplementary Information

Below is the link to the electronic supplementary material.

Supplementary file1 (PDF 1052 KB)
