# Supplementary material for: Enhancing the Interaction of Carbon Nanotubes by Metal–Organic Decomposition with Improved Mechanical Strength and Ultra-Broadband EMI Shielding Performance
Source: Nanomicro Lett. 2024 Feb 27;16:134. doi: 10.1007/s40820-024-01344-1 (PMC10899147; doi:10.1007/s40820-024-01344-1)
Supplement: Supplementary file 1 — Supplementary file1 (PDF 1052 KB) [file 40820_2024_1344_MOESM1_ESM.pdf]

Supporting Information for

Enhancing the Interaction of Carbon Nanotubes by Metal-Organic Decomposition with Improved Mechanical Strength and Ultra-Broadband EMI Shielding Performance

Yu-Ying Shi[1, 2, ‡], Si-Yuan Liao[1, ‡], Qiao-Feng Wang[1], Xin-Yun Xu[1], Xiao-Yun Wang[1], Xin-Yin Gu[1], You-Gen Hu[1, *], Peng-Li Zhu[1], Rong Sun[1], Yan-Jun Wan[1, 3, *]

[1]Shenzhen Institute of Advanced Electronic Materials, Shenzhen Institutes of Advanced Technology, Chinese Academy of Sciences, Shenzhen 518055, P. R. China

[2]Southern University of Science and Technology, Shenzhen 518055, P. R. China

[3]National Key Laboratory of Materials for Integrated Circuits, Shanghai Institute of Microsystem and Information Technology, Chinese Academy of Sciences, Shanghai 200050, P. R. China

‡Yu-Ying Shi and Si-Yuan Liao contributed equally to the work.

*Corresponding authors. E-mail: yg.hu@siat.ac.cn (You-Gen Hu); yj.wan@siat.ac.cn (Yan-Jun Wan)

Supplementary Figures and Tables

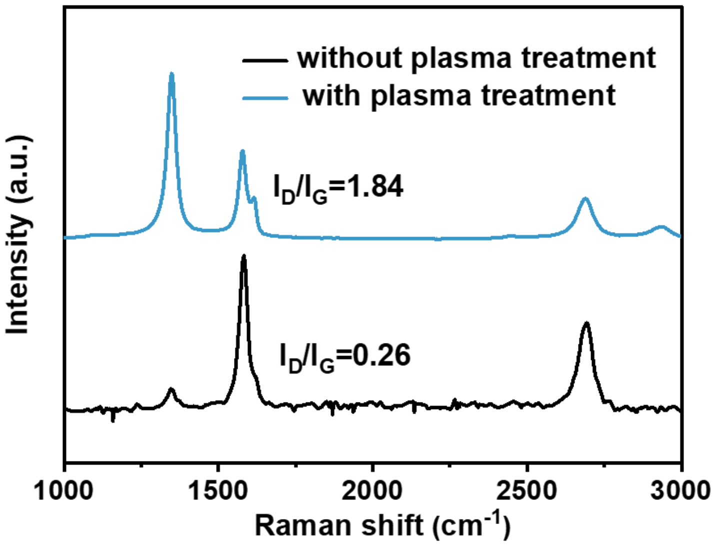

Fig. S1 Raman spectra of CNT films with and without plasma treatment

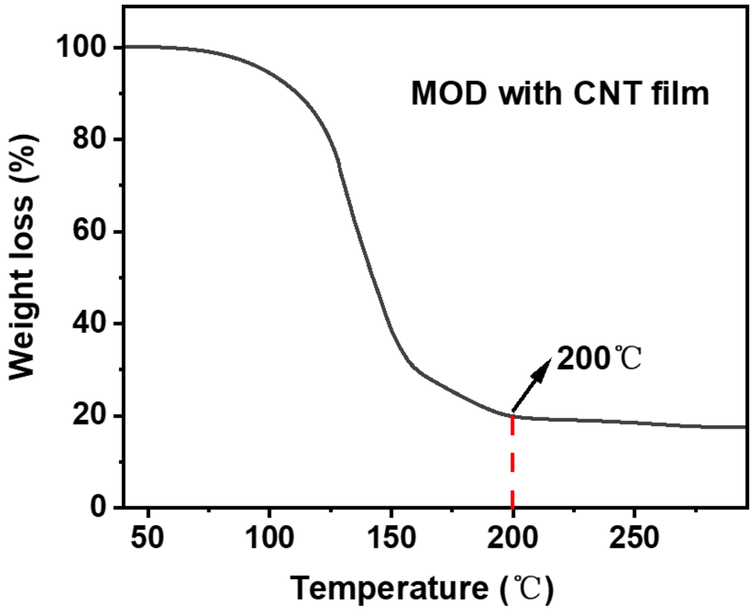

Fig. S2 Thermogravimetric analysis curves of the MOD with CNT film

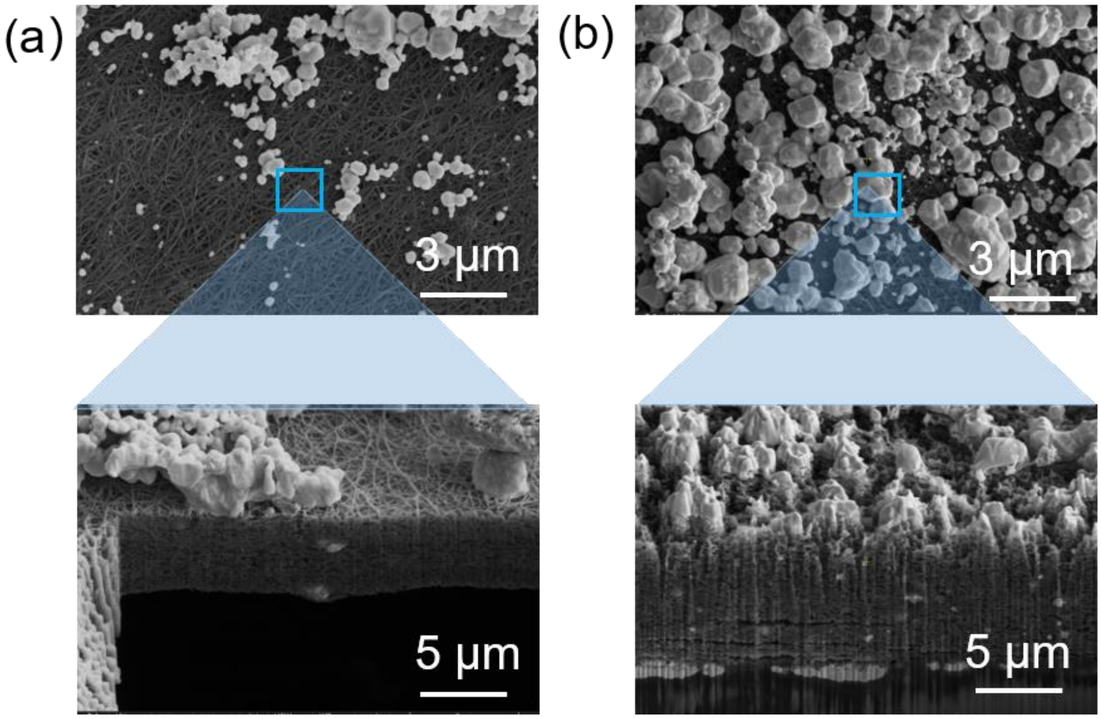

Fig. S3 SEM images of Ag-CNT film (**a**) without plasma treatment and (**b**) with plasma treatment

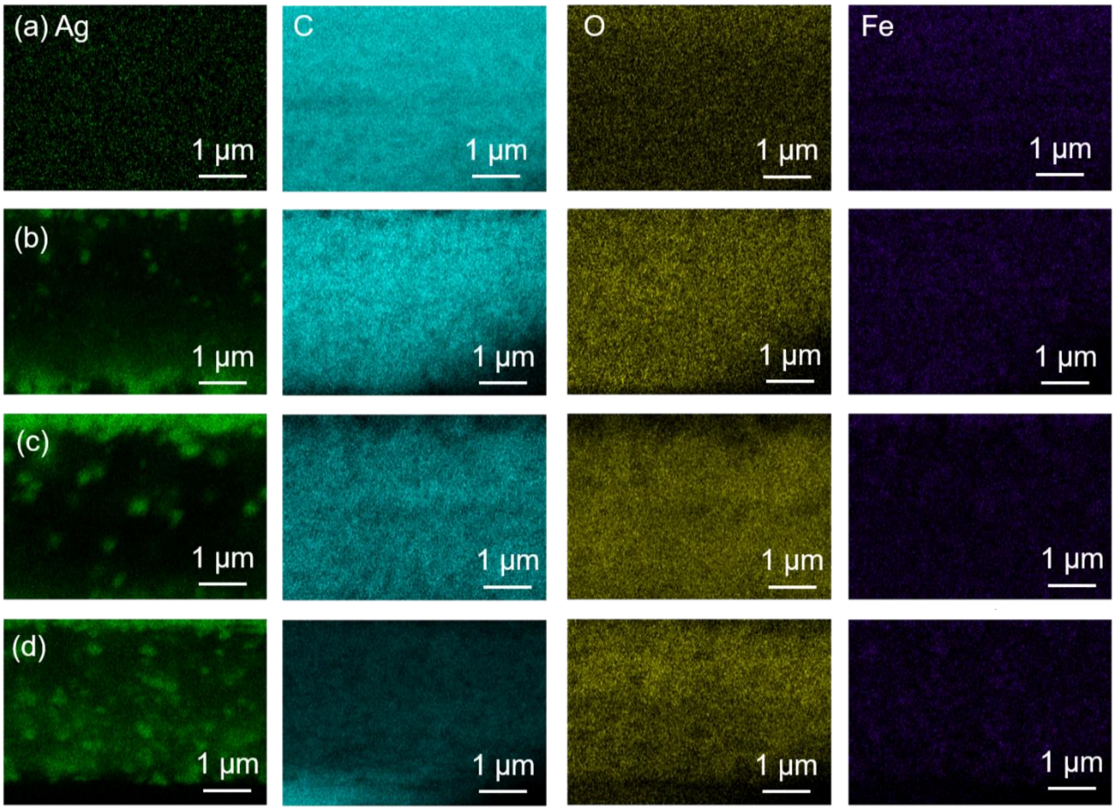

Fig. S4 The corresponding EDS maps of (**a**) CNT film. (**b**) Ag-CNT film-1. (**c**) Ag-CNT film-2. (**d**) Ag-CNT film-3

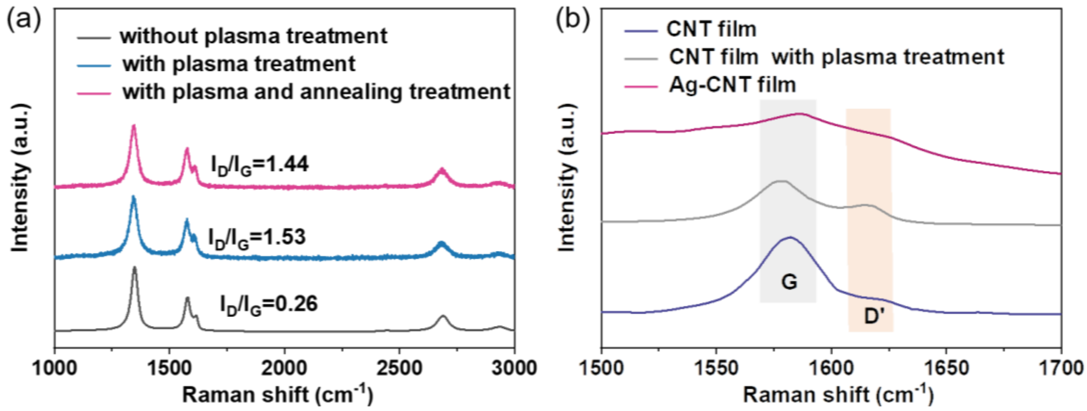

Fig. S5 (**a**) Raman spectra of CNT films. (**b**) Strong G peak with a small shoulder D' peak of CNT and Ag-CNT films

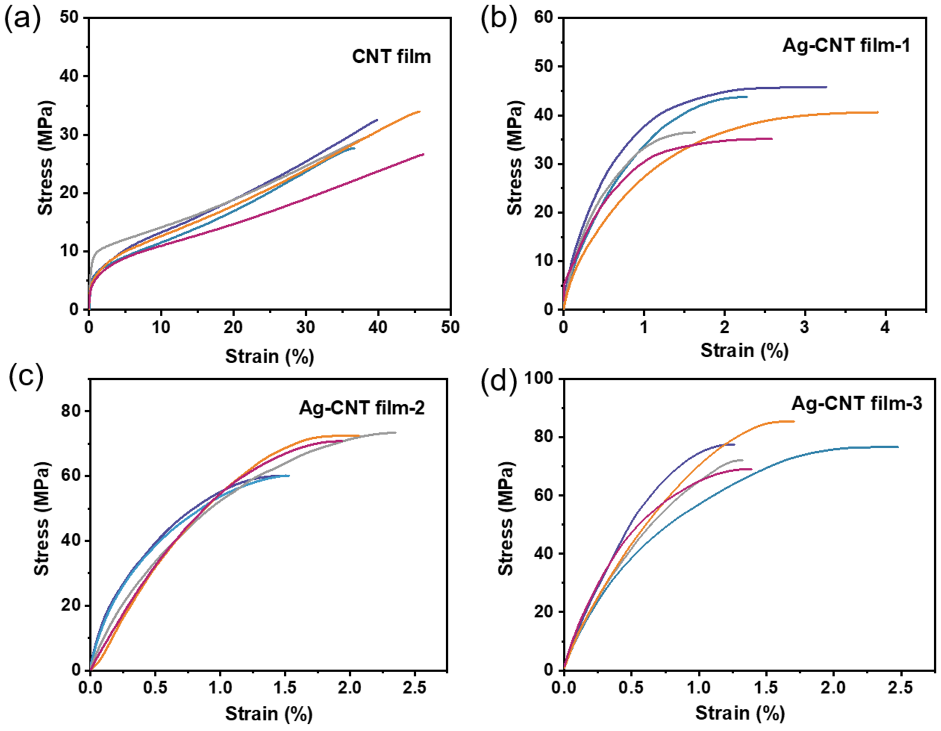

Fig. S6 (a-d) Stress-strain curves of CNT film and Ag-CNT films with different Ag content

Table S1 Summary of mechanical properties of CNT film and Ag-CNT films

Sample	Tensile strength (MPa)	Elongation at break (%)	Young's modulus (GPa)
**CNT film**	30.09±3.14	41.39 ± 4.30	1.12±0.33
**Ag-CNT-film-1**	40.38±4.56	2.73 ± 0.87	4.55±0.65
**Ag-CNT-film-2**	71.52±7.42	2.06 ± 0.73	7.04±0.71
**Ag-CNT-film-3**	76.06±6.20	1.63 ± 0.50	8.90±0.97

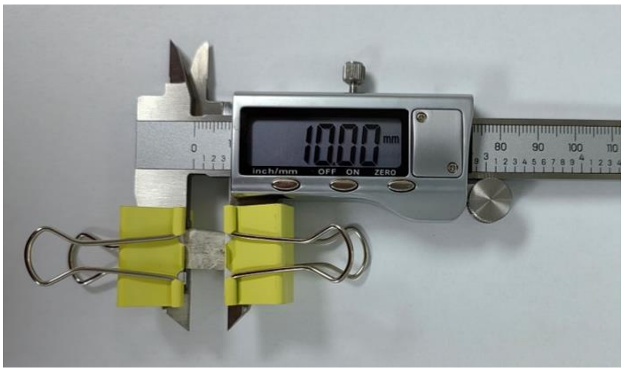

Fig. S7 Home-made devices for *in-situ* Raman test

Table S2 Summary of mass, thickness, electrical conductivity, and EMI SE of Ag-CNT films

**Sample**	**CNT film**	**Ag-CNT-film-1**	**Ag-CNT-film-2**	**Ag-CNT-film-3**
**Ag content (wt %)**	0	42	51	66
**Thickness (μm)**	5	5.9	6.5	7.8
**Electrical conductivity (S/m)**	77040	84960	324333	682000
**Average EMI SE (3 - 40 GHz, dB)**	37	41	48	66
**SSE (dB • cm^{-1})**	74000.0	69491.5	73846.2	84615.4

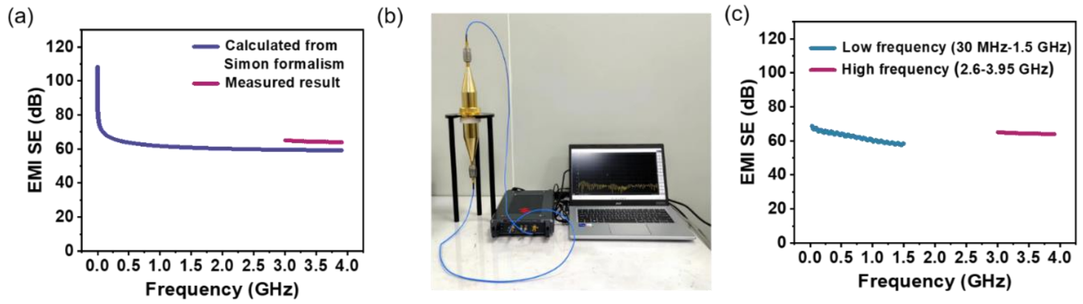

Fig. S8 (**a**) Experimental and theoretical EMI SE of Ag-CNT film. (**b**) Measuring equipment of shielding performance and (**c**) Experimental EMI SE measurements of Ag-CNT film, show similar EMI SE values at lower and higher frequencies

The theoretical EMI SE values derived from Simon's formula over a broad frequency range are compared with the experimental values in the S-band (Figure S8a). Calculated results predict high EMI SE values at low frequencies as well. According to ASTM D4935-99 standard, the EMI SE in the frequency range of 30 MHz - 1.5 GHz is tested by coaxial transmission line method. The measurement set-up consisted of a sample holder (KEYCOM, Japan) with its input and output connected to the network analyzer (Figure S8b). The SE values of the films in the low-frequency range were evaluated, and the results are shown in Figure S8c.

Equation part

(1) The input impedance of a single-layer shield (Z) can be calculated according to the following equation [S1]:

$$Z = Z_0 \left(\frac{\mu_r}{\varepsilon_r}\right)^{\frac{1}{2}} \tanh\left(\frac{j(2\pi f d)(\mu_r \varepsilon_r)^{\frac{1}{2}}}{c}\right) \quad \text{(S1)}$$

(2) The reflection loss of the shielding surface from front to back can be calculated

using the following formula [S2]:

$$SE_R = 20\log\frac{(Z+Z_0)^2}{4ZZ_0} = 39.5 + \log\left(\frac{\sigma}{2f\pi\mu}\right) \quad \text{(S2)}$$

(3) The absorption loss of shielding material can be expressed as follows [S3]:

$$SE_A = 20\left(\frac{d}{\delta}\right)\log e = 8.68\left(\frac{d}{\delta}\right) = 8.68\frac{\sqrt{f\mu\sigma}}{2} \quad \text{(S3)}$$

where Z_0, ε_r, and ε_r are the impedances of in free space, relative complex permittivity, and relative complex permeability, respectively. As Z approaches Z_0, the impedance matching between free space and the shield improves, allowing EMWs to penetrate more into the shielding material. f and c are the frequency and velocity of the EMWs. d, σ and μ are the thickness, electrical conductivity, and magnetic permeability of the shield, respectively.

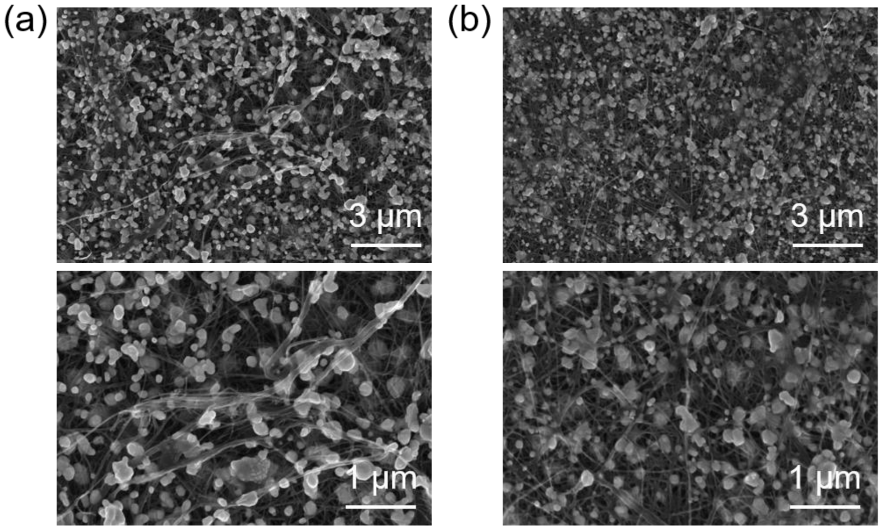

Fig. S9 The SEM images of Ag-CNT films (**a**) before and (**b**) after 2000-cycle bending

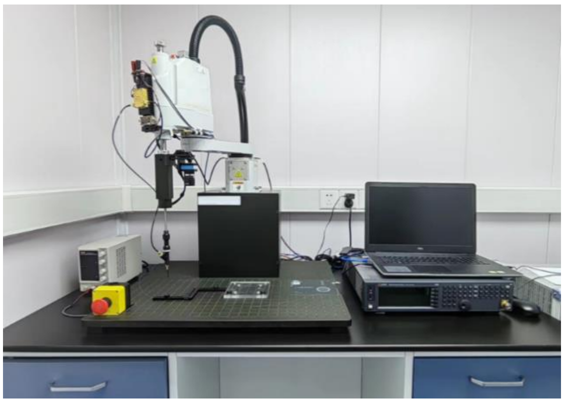

Fig. S10 Measuring equipment of near-field shielding performance

The near-field radiation is typically dominated in the region of $KR \ll 1$. The delay between phase and energy propagation of the EM waves can be ignored in this case, and the near-field radiation can be served as a quasi-static condition. To accurately measure the near-field SE of Ag-CNT film, a microstrip antenna embedded in a printed

circuit board serves as an analog chip and used as a near-field EM radiation source[4]. The scanning probe connected to VNA via a coaxial cable with an SMA connector is employed as the signal collector, as shown in Figure S10. The probe directly measures the electromagnetic wave radiation intensity at a specific point in space, while the shielding efficiency of the material is determined by comparing the radiation intensity before and after shielding.

Supplementary References

[S1] M. Panahi - Sarmad, S. Samsami, A. Ghaffarkhah, S. A. Hashemi, S. Ghasemi et al., MOF-based electromagnetic shields multiscale design: nanoscale chemistry, microscale assembly, and macroscale manufacturing. Adv. Funct. Mater. 2023, 2304473. https://doi.org/10.1002/adfm.202304473

[S2] A. Iqbal, P. Sambyal and C. M. Koo, 2D MXenes for Electromagnetic Shielding: A Review. Adv. Funct. Mater. 2020, **30**, 2000883. https://doi.org/10.1002/adfm.202000883

[S3] S. A. Hashemi, A. Ghaffarkhah, E. Hosseini, S. Bahrani, P. Najmi et al., Recent progress on hybrid fibrous electromagnetic shields: Key protectors of living species against electromagnetic radiation. Matter 2022, **5**, 3807-3868. https://doi.org/10.1016/j.matt.2022.09.012

[S4] Y. Xu, Z. Lin, K. Rajavel, T. Zhao, P. Zhu et al., Tailorable, lightweight and superelastic liquid metal monoliths for multifunctional electromagnetic interference shielding. Nano-Micro Lett. 2022, **14**, 29. https://doi.org/10.1007/s40820-021-00766-5
